# IgE glycans promote anti-IgE IgG autoantibodies that facilitate IgE serum clearance *via* Fc Receptors

**DOI:** 10.3389/fimmu.2022.1069100

**Published:** 2022-12-02

**Authors:** Kevin Plattner, Zahra Gharailoo, Simon Zinkhan, Paul Engeroff, Martin F. Bachmann, Monique Vogel

**Affiliations:** ^1^ Department of Immunology, University Hospital for Rheumatology and Immunology, Bern, Switzerland; ^2^ Department of Biomedical Research (DBMR), University Clinic for Rheumatology and Immunology, University of Bern, Bern, Switzerland; ^3^ Nuffield Department of Medicine, The Jenner Institute, University of Oxford, Oxford, United Kingdom

**Keywords:** allergy, IgG anti-IgE autoantibodies, glycans, hypersensitivity, IgE regulation

## Abstract

**Background:**

Recent studies have shown that IgE glycosylation significantly impacts the ability of IgE to bind to its high-affinity receptor FcεRI and exert effector functions. We have recently demonstrated that immunizing mice with IgE in a complex with an allergen leads to a protective, glycan-dependent anti-IgE response. However, to what extent the glycans on IgE determine the induction of those antibodies and how they facilitate serum clearance is unclear.

Therefore, we investigated the role of glycan-specific anti-IgE IgG autoantibodies in regulating serum IgE levels and preventing systemic anaphylaxis by passive immunization.

**Methods:**

Mice were immunized using glycosylated or deglycosylated IgE-allergen-immune complexes (ICs) to induce anti-IgE IgG antibodies. The anti-IgE IgG antibodies were purified and used for passive immunization.

**Results:**

Glycosylated IgE-ICs induced a significantly higher anti-IgE IgG response and more IgG-secreting plasma cells than deglycosylated IgE-ICs. Passive immunization of IgE-sensitized mice with purified anti-IgE IgG increased the clearance of IgE and prevented systemic anaphylaxis upon allergen challenge. Anti-IgE IgG purified from the serum of mice immunized with deglycosylated IgE-ICs, led to a significantly reduced elimination and protection, confirming that the IgE glycans themselves are the primary drivers of the protectivity induced by the IgE-immune complexes.

**Conclusion:**

IgE glycosylation is essential for a robust anti-IgE IgG response and might be an important regulator of serum IgE levels.

## 1 Introduction

IgE and its effector cells, such as basophils and mast cells, help eradicate pathogens, like parasites and helminths, and degrading venoms (1). However, type I hypersensitivity disorders are also IgE-mediated. IgE binds with high affinity to the FcεRI receptor on effector cells. The cross-linking of bound IgE antibodies by antigens leads to the activation and degranulation of effector cells, resulting in an inflammatory response and the associated typical allergic symptoms (2, 3). Since approximately one-third of the world’s population suffers from allergic disease, it is crucial to know how levels of IgE are regulated (4). Unfortunately, the mechanisms of how the body regulates IgE are poorly understood.

IgE’s shorter serum half-life of 2-2.5 days, compared to serum IgG, which has a half-life of roughly three weeks in humans, is a crucial aspect of IgE control (5, 6). In addition, it has been demonstrated that the IgE Fc receptors FcεRI and FcεRII (CD23) control the levels of IgE in the serum (7, 8). Additionally, the synthesis of new IgE antibodies is negatively regulated by CD23 on B cells (9, 10). IgE is the most glycosylated antibody, but IgE-glycans’ role in their serum regulation is poorly understood. Glycosylation, required for antibody maturation and effector function, has been shown to affect IgE function. Crystal structures of human IgEs have revealed seven and nine N-glycosylation sites for human and mice constant epsilon chains, respectively (11). Recently, Shade et al. have provided insights into the role of IgE glycosylation in allergic diseases. They showed that IgE from peanut-allergic individuals has more sialic acid than non-atopic individuals and that removal of sialic acids leads to an attenuation of effector cell degranulation, contributing thereby to the effector function of IgE (12). The same group also demonstrated that a conserved oligomannose attached to N394 on the human Cε3 domain is essential for binding to the FcεRI (13).

In mice, the equivalent of N394 is N384. Consistent with human findings, IgE mutated or lacking oligomannose could not elicit an anaphylactic reaction in a passive cutaneous anaphylaxis mouse model (13). In addition to endogenous regulation, external factors like anti-IgE IgG antibodies also influence IgE levels. For example, the monoclonal anti-IgE antibody omalizumab got approval for moderate to severe persistent asthma, chronic idiopathic urticaria, and nasal polyps and has shown clinical efficacy in these diseases (14–19). Omalizumab might also be effective in other allergic conditions like food allergies (20, 21). Mechanistically, omalizumab acts primarily by neutralizing free serum IgE but also disrupts IgE : FcεRI complexes (22, 23). As a result, less IgE is bound by FcεRI on effector cells over time, preventing their IgE-dependent activation.

Interestingly, the body can produce anti-IgE IgG antibodies itself. Atopic and healthy individuals express endogenous anti-IgE IgG antibodies, which may define another way of IgE regulation (24, 25). Although the presence of these antibodies has been known for a long time, their function is still enigmatic. Anti-IgE IgG antibodies can potentially activate effector cells by cross-linking FcεRI-bound IgE and thus lead to an anaphylactic reaction (26). On the other hand, they also have beneficial effects by reducing type I allergic reactions. The induction of anti-IgE antibodies significantly suppresses total and antigen-specific IgE responses (27–30). CD23 may also play a role in the mechanism of action of IgG and IgE antibodies. Indeed, IgE immune complexes bind better to CD23 than IgE alone (31), which might also increase the protection of CD23 from being enzymatically cleaved (32). Recently, our group has shown that immunizing mice with IgE in a complex with an allergen can induce glycan-dependent anti-IgE IgG antibodies. These mice were protected from challenges with different allergens (33). However, it was unclear whether glycans on IgE antibodies are responsible for that protection and how they promote IgE clearance. Here we performed passive immunization with glycan-specific and non-glycan-specific anti-IgE IgG autoantibodies and investigated differences between anti-IgE IgG antibodies in IgE clearance and protection upon allergen challenges, concluding that glycans are critical for anti-IgE IgG mediated protection.

## 2 Materials and methods

### 2.1 Allergens and allergen-specific monoclonal antibodies

The production of recombinant Fel d 1 and monoclonal Fel d 1-specific IgE F127 antibody used in these experiments have been described previously (34).

Briefly, the sequence encoding a fusion of chains 1 and 2 of Fel d 1 spaced by a 15aa-linker (GGGGS) 3 was linked to a histidine tag and then cloned into the plasmid pET42 (Addgene, Watertown, Mass). Recombinant Fel d 1 was generated at 20°C for 20 hours after the plasmid was transformed into Escherichia coli C25661 (New England Biolabs, Ipswich, Massachusetts). A Ni2+ affinity column purified the supernatant after the cells had been sonicated. Size-exclusion chromatography was used with a Superdex 75 column (GE Healthcare, Chicago, Illinois) to separate monomers and dimers from multimers.

The monoclonal IgE F127 was produced in CHO cells (Evitria AG, Zürich, Switzerland) and purified by affinity chromatography over a protein L or protein G Sepharose column (GE Healthcare), respectively.

The anti-Ara h2 IgE sequence (P12P3D08) was obtained from Croote et al. (35). It was expressed as mouse IgE and produced in HEK cells using Expifectamine^®^ (Thermo Fisher Scientific, Waltham, MA, USA) and purified by immunoaffinity using a CaptureSelect XP C‐tag column (Thermo Fisher).

Roasted peanut extract (Ara R) was prepared according to the protocol of Koppelman et al. (36)

Antibodies were deglycosylated using PNGase F deglycosylation kit (Thermo Fisher) under native conditions according to the supplier’s protocol, except that the protein was deglycosylated at 37°C and overnight. Zeba™ Spin Desalting Columns (MWCO 40K MWCO; Thermo Fisher) were used to purify the antibodies. PNGase F treated IgE is from now on called IgE(PNG).

### 2.2 Mouse immunization and sampling

Mice were kept at the central animal facility (Murtenstrasse 31, Bern, Switzerland) and used for an experiment between the ages of 8 and 12 weeks. BALB/c mice were purchased from Envigo (Envigo, Huntingdon, UK). The Swiss Federal Veterinary Office approved all protocols used to treat experimental animals. Prof. J. Ravetch kindly provided CD23-/- mice. FcγRIIb -/- and Fcγ common chain KO (FcγR KO) mice (Jackson Laboratory, Bar Harbor, Maine, MA, USA) were purchased at 6 weeks and bred in our facility. All animals were acclimatized to the facility for at least one week. IgE-Fel d 1 complexes were formed for immunizations by incubating 25μg IgE and 5μg Fel d 1 for 1h at 37°C dissolved in 100μl PBS before intravenous (i.v.) injection into mice. To analyze IgE clearance, mice were injected with or without 25μg anti-IgE IgG or anti-IgE(PNG) IgG 1h before receiving 25μg IgE intravenously. Blood from tail veins was collected using Microtainer^®^ serum tubes (BD Biosciences, Franklin Lakes, NJ, USA) or in PBS containing 10mM EDTA (Sigma-Aldrich, St Louis, Mo, USA). Blood was collected from naïve mice and from IgE injected mice 30 minutes, 2 hours, and 24 hours after injection.

### 2.3 Fluorospot analysis

Fluorospot assay was performed according to the manufacturer’s protocol. The Fluorospot plate (Mabtech) was coated with 50μg/mL IgE per well at 4°C overnight. First, 1x10^6^ cells from BM and spleen from triple immunized mice were seeded per well. The plate was incubated at 37°C (5% CO_2_) for 20h. Next, goat anti-mouse IgG biotin primary Ab (SouthernBiotech, Birmingham, AL, USA) diluted in PBS-0.1% BSA (1:1000) was incubated for 2h at RT. Then, streptavidin-550 (Mabtech) diluted in PBS-0.1% BSA (1:200) was added for 1h at RT. Next, fluorescence enhancer-II (Mabtech) was added for 15 min at RT. The plate was dried at RT overnight. Finally, the plate was read at 550nm using the Fluorospot reader (Mabtech IRIS).

### 2.4 IgG isolation and purification

According to the manufacturer’s manual, total IgG was purified by Protein G column chromatography (HiTrap Protein G HP, 1ml, Cytiva, Marlborough, MA, USA) from pooled sera (5ml) obtained by terminal bleeding. For isolating Fel d 1-specific IgG, Fel d 1 was coupled to the HiTrap NHS-Activated HP 1ml column (Cytiva) according to the manufacturer’s manual. The total IgG was applied. The flow-through and the eluate were collected. Buffer exchange to PBS was performed using Vivaspin^®^ 6 10 kDa MWCO (Cytiva).

### 2.5 Flow cytometry

Red blood cells were lysed using ACK buffer (Thermo Fisher). Unspecific binding was blocked by using mouse Fc gamma block (BD Bioscience). Basophils were marked with APC anti-mouse CD49b (clone HMα2, Biolegend) and PE anti-mouse IgE (clone RME-1, Biolegend). Antibody staining were carried out at 4°C for 30min. After the staining, the cells were washed 3x with PBS. Flow cytometry was performed with CytoFLEX S 4L 13C (B2-R3-V4-Y4) plus 96 DW plate loader (Beckman Coulter Life Sciences, CA, USA) and analyzed using FLOWJO software (TreeStar Inc, Ashland, OR, USA).

### 2.6 Passive immunization and challenge

Mice were injected with 25µg anti-IgE IgG, 25µg anti-IgE(PNG) IgG, or 25µg control IgG (Purified mouse IgG1, k, MOPC-31C, BD Bioscience) in 100μl PBS i.v. 24 hours later, mice were sensitized with 5µg IgE F127 or 5µg anti-Ara h2 IgE in 100µl PBS. The following day, the MiniTemp rectal probe for mice (Vetronic Services Ltd, Abbotskerswell, UK) was used to measure the baseline body temperature. Rectal temperature was monitored every ten minutes for an hour after intravenous injections of 20µg of roasted peanut extract (Ara R) or 5µg of Fel d 1 in IgE-sensitized mice.

### 2.7 Elimination of IgE-anti-IgE IgG complexes *in vitro*

Primary mouse B cells were isolated from the spleen of Balb/c and CD23 KO mice. The B cells were isolated using EasySep™ Mouse B cell isolation Kit (STEMCELL Technologies, Vancouver, Canada) according to the manufacturer’s manual. The B cells were kept in RPMI1460 medium with 10% FBS at a density of 100’000 cells in 100µl. IgE anti-IgG complexes were formed by incubating 2µg/ml of 4µg/ml of anti-IgE IgG in medium for 1h at 37°C. Next, the medium of the B cells was removed and 5µl of complexes were added per well. Supernatant was taken and used for ELISA after 10min and 30min of incubation time. After removing the supernatant, the cells were immediately stained with rat anti-mouse IgE-FITC (Clone R35-72 (RUO), BD Bioscience) and goat anti-mouse IgG-APC (ThermoFisher Scientific) as described above. The cells were washed twice with PBS and fixed with 4% formaldehyde in PBS for 15min at room temperature. Flow cytometry was performed as described above.

### 2.8 ELISA assays

96-well Nunc Maxisorp™ ELISA plates (Thermo Fisher) were coated with 2µg/ml antibody in PBS at 4°C overnight. After blocking with PBS/0.15% Casein solution for 2h at room temperature, plates were washed 5x with PBS. Incubation of antibodies or sera was performed for 1h at RT. Afterward, plates were washed 5x with PBS/0.05% Tween. ELISAs were developed with TMB (3,30,5,50-tetramethylbenzidine) and H_2_O_2_ and stopped with 1mol/L sulfuric acid after 10 minutes. OD was measured at 450 nm.

#### 2.8.1 Detection of mouse IgG specific to IgE, IgE(PNG), and Fel d 1 and their subclasses after immunization with IgE-ICs

Plates were coated with mouse IgE F127, IgE(PNG), or Fel d 1. Serial dilution of sera (1:25, then serially 1:3) was added. HRP-labeled goat anti-mouse IgG (The Jackson Laboratory) antibodies were used to detect IgG (1:2’000). To detect IgG subclasses, HRP labeled rat anti-mouse IgG1 (BD Pharmingen), goat anti-mouse IgG2a (Bio-Rad), goat anti-mouse IgG2b (Invitrogen, Carlsbad, CA, USA), and goat anti-mouse IgG3 (SouthernBiotech, Birmingham, AL, USA) were used in a 1:2’000 dilution.

#### 2.8.2 Assay for assessing IgE and IgE-IgG complexes clearance in serum and *in vitro*

Anti-mouse IgE STAR110 (Bio-Rad) was coated. Then, serial dilution of sera (1:25, then 1:3 serially), or supernatant from the B cells were added. Goat anti-mouse IgE-HRP STAR110P (Bio-Rad) was used to detect mouse IgE (1:2’000), and HRP-labeled anti-mouse IgG was used to detect IgE-IgG complexes (1:2’000).

#### 2.8.3 Assessment of anti-IgE IgG purification

Plates were coated with IgE, IgE(PNG), or Fel d 1. The eluate and flow-through of the protein G and Fel d 1-column were tested starting with 2μg/ml and further serially diluted 1:3. HRP-labeled goat anti-mouse IgG (The Jackson Laboratory) antibodies were used for detection of IgG (1:2’000).

#### 2.8.4 Avidity ELISA

IgE F127 was coated. Isolated anti-IgE IgG or anti-IgE(PNG) were applied at 2μg/ml and were further serial diluted (1:3). After 1h incubation, the plates were washed 3 times for 5min either with 7M urea in PBST (PBS mixed with 0.05% Tween20) or PBST only. To detect mouse IgG antibodies, HRP-labeled goat anti-mouse IgG (The Jackson Laboratory) was used. The formula used to determine the avidity index (AI) was AIx = OD (dilution x) + urea/OD (dilution x)–urea.

### 2.9 Statistical analysis

Statistics were carried out using GraphPad Prism 9.0 (GraphPad Software, Inc, San Diego, CA, USA). To determine the statistical significance between two groups, a student’s t-test was used. More than two groups were examined by one-way ANOVA. Two-way ANOVA was used to compare variables that depended on concentration and time, and then Tukey testing was applied. All data are presented as mean SEM in graphs. The signs for statistical significance are ns = not significant, p ≤ 0.05 (*), p ≤ 0.01 (**), p ≤ 0.001(***), and p ≤ 0.0001 (****).

## 3 Results

### 3.1 Higher levels of anti-IgE IgG antibodies are induced by immunization with IgE-Fel d 1 complexes compared to complexes containing deglycosylated IgE

**Figure 1:**
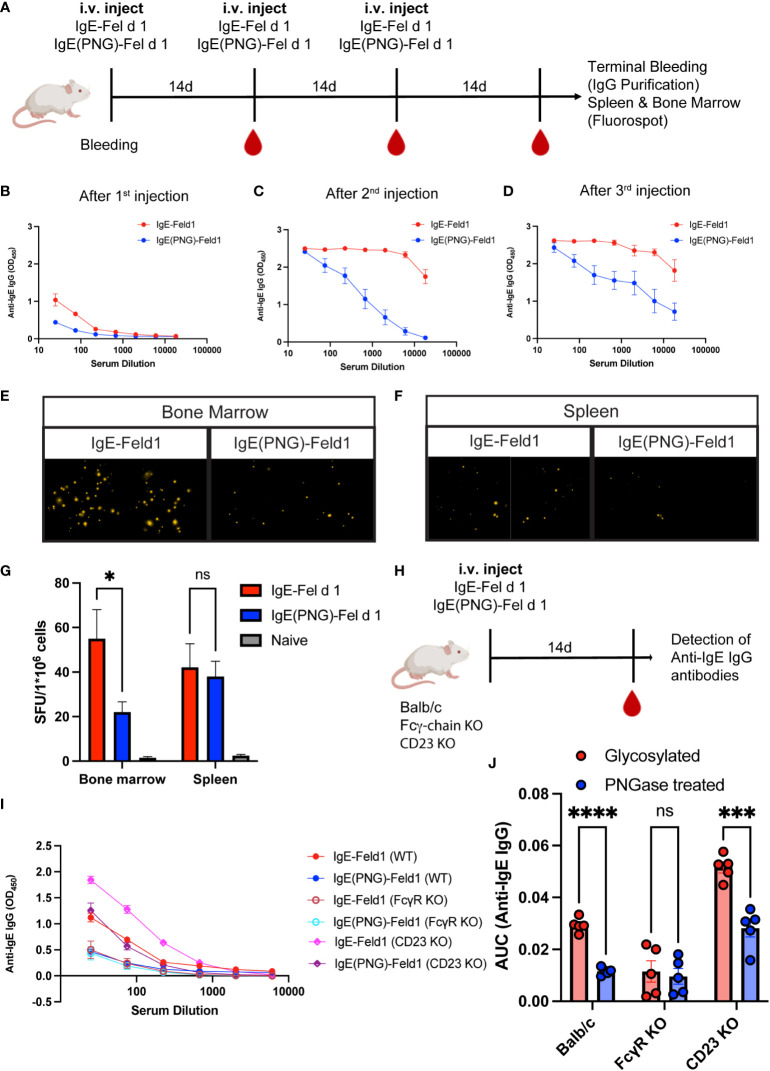
Glycosylated IgE in complex with Fel d 1 induces more IgG antibodies against IgE than deglycosylated IgE-Fel d 1 complexes. Schematic overview of the immunization regiment and timepoints of experiments **(A)**. Anti-IgE IgG response (at OD450 by ELISA) **(B-D)** 14 days after first immunization with IgE-Fel d 1 or IgE(PNG)-Fel d 1 **(B)**, after the second immunization **(C)**, and after third immunization **(D)**. Representative images of fluorospot experiments of anti-IgE IgG producing B cells **(E, F)** from Bone marrow **(E)** or spleen **(F)**. Spot forming units per 1 million cells in bone marrow and spleen **(G)**. Schematic overview of the immunization of Balb/c, CD23KO and Fcγ-chainKO mice to compare the anti-IgE IgG response **(H)**. Anti-IgE IgG response at OD450 by ELISA **(I)** or as AUC **(J)** of Balb/c, CD23KO and Fcγ-chainKO mice 14 days after immunization with IgE-IC or IgE(PNG)-IC. Statistical analysis (mean ± SEM) using unpaired students t-test **(G)**, n = 6, and multiple paired t-test **(J)**, n=5. The signs for statistical significance are ns = not significant, p ≤ 0.05 (*), p ≤ 0.001 (***), and p ≤ 0.0001 (****).

We first investigated the IgE-specific IgG response induced by immunization with glycosylated (IgE-ICs) or deglycosylated (IgE(PNG)-IC) IgE in a complex with Fel d 1 (**Figure 1A**). Serum anti-IgE IgG antibodies were measured by ELISA (**Figures 1B–D**). Both groups generated antibodies against IgE after immunization, which increased following the first and the second booster. However, mice immunized with IgE-ICs showed a higher anti-IgE IgG response than mice immunized with IgE(PNG)-IC. The IgG response against Fel d 1 and IgE(PNG) also increased after each boost, and the response was consistently higher in the group receiving IgE-ICs (**Supplementary Figures 1A–D**).

IgE-specific plasma cells were quantified in lymphoid organs upon IgE-ICs or IgE(PNG)-ICs vaccination. For that, spleen and bone marrow (BM) were collected two weeks after the third injection (day 42) and analyzed for the presence of IgE-specific IgG-secreting plasma cells. Bone marrow and spleen contained anti-IgE IgG-secreting plasma cells (**Figures 1E, F**). On average, 55 IgG-secreting plasma cells were found per million bone marrow cells in the mice immunized with IgE-ICs compared to 22 in the deglycosylated group. In contrast, in the spleens of mice immunized with glycosylated and deglycosylated IgE, a similar number of IgG-secreting plasma cells were found (**Figure 1G**). The reduced levels of plasma cells, together with reduced avidities of the antibodies (see below), may explain the observed differences in antibody titers.

In addition, we measured the elimination of IgE, IgE(PNG), IgE-IC, and IgE(PNG)-IC from serum, and observed that IgE disappears from the serum faster than IgE(PNG). Furthermore, compared to free IgE antibodies, IgE-IC are eliminated more quickly, whereby no difference was found between glycosylated and deglycosylated complexes (**Supplementary Figure 2A**).

Next, we investigated whether IgE’s glycosylation influences anti-IgE IgG subclasses’ formation. As in our previous study (33), IgG1 and IgG2b were the most frequent subclasses. Additionally, no significant difference between the glycosylated and deglycosylated groups was detectable (**Supplementary Figure 2B**).

As we previously showed that IgE-IC binding to B cells was CD23 dependent we assessed the role of CD23 in generating anti-IgE IgG by immunizing CD23 KO mice once with IgE-IC and IgE(PNG)-IC (**Figure 1H**). Compared with WT mice, CD23 KO mice showed after two weeks immunization higher anti-IgE IgG levels supporting the hypothesis that CD23 might act as a regulator for the production of anti-IgE antibodies (31), However, as for WT mice higher level of anti-IgE IgG was obtained with glycosylated than with deglycosylated IgE-IC. Further, we also looked at the role of FcγR and FcϵRI using Fcγ common chain KO (lacking FcεRI, FcγRI, and FcγRIIIa) in mediating anti-IgE IgG and showed that KO mice immunized with IgE-IC have lower anti-IgE IgG levels than WT mice. As shown in **Figures 1I, J** this was independently of whether IgE-IC is glycosylated or not suggesting that IgE-ICs binding is not only CD23 dependent.

In conclusion, triple vaccination with IgE-ICs without adjuvant elicited a solid systemic humoral immune response against IgE, which was significantly stronger when glycosylated IgE was used as the immunogen. These results indicate that glycans play a role in modulating anti-IgE IgG responses that are probably FcγR dependent.

### 3.2 Enhanced serum IgE clearance by anti-IgE IgG compared to anti-IgE(PNG) IgG

We next wanted to investigate the effects of anti-IgE IgG and anti-IgE(PNG) IgG antibodies on the serum clearance of secondarily administered IgE and their ability to prevent sensitization of effector cells. Therefore, we isolated IgG antibodies from pooled sera by Protein G chromatography and analyzed them by ELISA. As expected, these antibodies reacted against IgE and Fel d 1 (**Supplementary Figure 3A** for IgE-Fel d 1, and **Supplementary Figure 3D** for IgE(PNG)-Fel d 1). Thus, to remove anti-Fel d 1-specific antibodies, the IgG antibodies were separated using a Fel d 1-coupled column. ELISA results of the eluate fraction show successful removal of Fel d 1-specific IgG antibodies (**Supplementary Figures 3B, 3E** for IgE and IgE(PNG) specific IgG; **Supplementary Figures 3C, 3F** for Fel d 1 specific IgG). From now on, the anti-IgE IgG isolated from mice vaccinated with IgE-ICs will be called anti-IgE IgG, and the ones from mice immunized with IgE(PNG)-ICs will be called anti-IgE(PNG) IgG.

**Figure 2:**
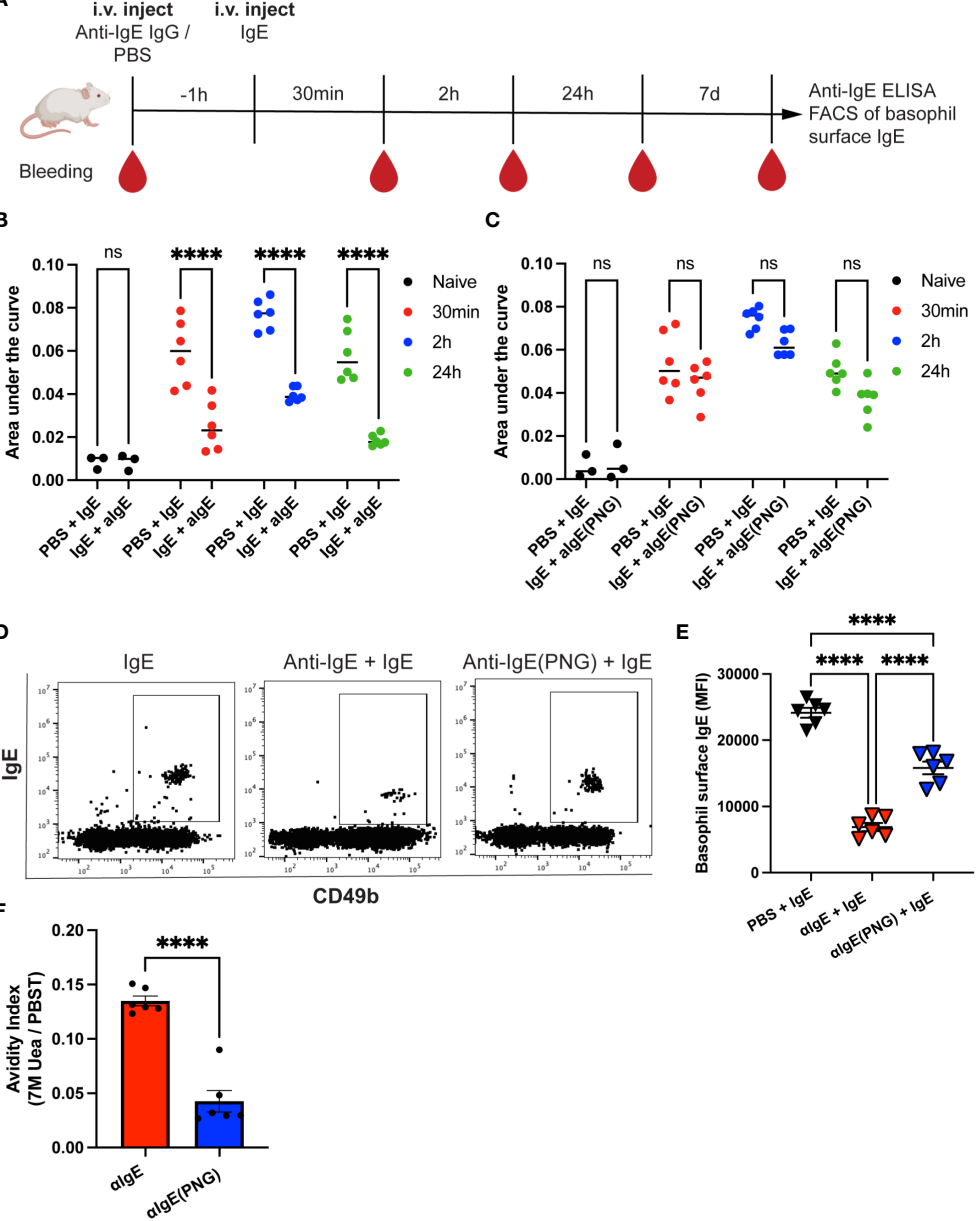
Anti-IgE IgG antibodies are more effective in lowering serum IgE levels than anti-IgE (PNG) IgG antibodies. Timetable with timepoints of injections, blood withdrawal, and experiments **(A)**. IgE serum levels were detected by ELISA and displayed as AUC at different time points after injection of IgE with or without passive immunization with anti-IgE IgG **(B, C)**. Shown are the results for IgE alone compared to IgE with anti-IgE IgG **(B)** and IgE alone compared to IgE with anti-IgE(PNG) IgG **(C)**. Representative FACS dot plot of basophils in mice immunized with IgE alone or with anti-IgE IgG or anti-IgE(PNG) **(D)**. Mean (± SEM) of IgE mean fluorescence intensity on basophils from naive mice, mice injected with IgE, and mice injected with anti-IgE IgG and IgE **(E)**. Avidity index of anti-IgE IgG and anti-IgE(PNG) to IgE **(F)**. Statistical analysis (mean ± SEM) using two-way ANOVA **(B, C)**, one-way ANOVA **(E)**, and unpaired students t-test **(F)**. n = 6 from two independent experiments. The signs for statistical significance are ns = not significant and p ≤ 0.0001 (****).

The purified anti-IgE IgG or anti-IgE(PNG) IgG antibodies were injected into mice 1h before IgE administration. Serum IgE was measured 30min, 2h, and 24h after IgE injection by ELISA. Additionally, Basophil surface IgE was measured one week after IgE injection (see the outline in **Figure 2A**). Anti-IgE IgG significantly increased the clearance of passively administered IgE in serum (**Figure 2B**). In contrast, using anti-IgE(PNG) IgG did not lead to a significant reduction in serum IgE levels (**Figure 2C**). Flow cytometry evaluated IgE sensitization of basophils one week after administration of IgE. The anti-IgE IgG group showed significantly decreased IgE surface levels compared to the IgE-only control group. The anti-IgE(PNG) IgG group also showed decreased IgE levels on basophils. However, their levels were significantly higher than the IgE levels of the anti-IgE IgG group (**Figures 2D, E**). Avidity ELISA was performed to analyze the avidity of the anti-IgE IgG antibodies. The results show that approximately 13% of the anti-IgE IgG and 4% of the anti-IgE(PNG) IgG have high avidity (**Figure 2F**). This difference could explain why anti-IgE IgG are better able to clear IgE from serum than anti-IgE(PNG) IgG.

These experiments conclude that anti-IgE IgG antibodies induced by glycosylated IgE are more efficient in downregulating passively administered IgE.

### 3.3 Anti-IgE IgG provides better protection against allergen challenges than anti-IgE(PNG) IgG

**Figure 3:**
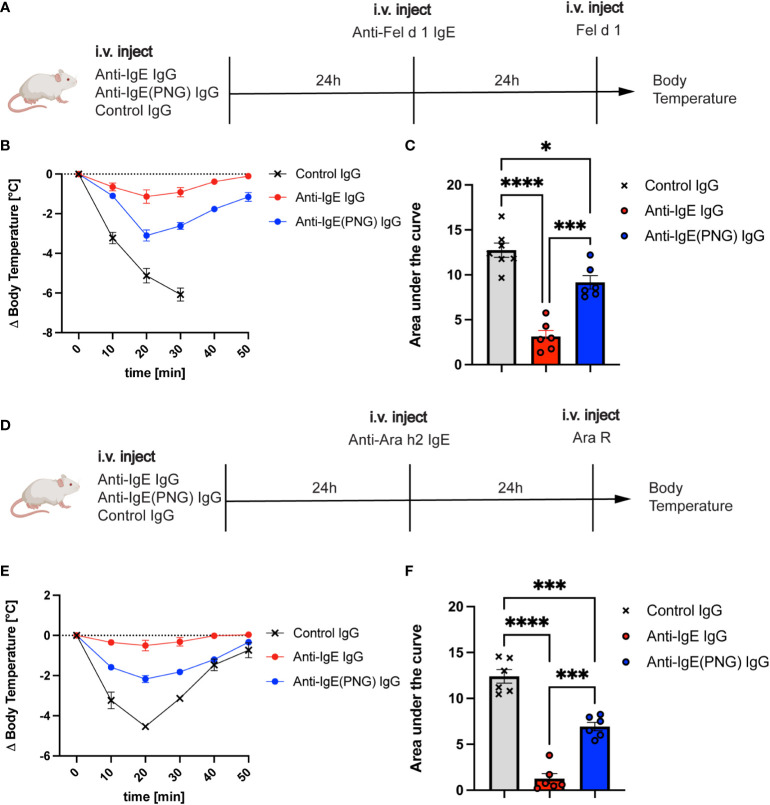
Passive immunization with anti-IgE-IgG protects against allergen challenges. The schematic overview of challenges with Fel d 1 and Ara R as an allergen in Balb/c mice **(A, D)**. Decrease in body temperature (delta) over time following challenge with Fel d 1 after passive immunization with anti-IgE IgG, anti-IgE(PNG) IgG, or control IgG **(B)**. The area under the curve of the challenge with Fel d 1 **(C)**. Decrease in body temperature (delta) over time upon challenge with Ara R after passive immunization with anti-IgE IgG, anti-IgE(PNG) IgG, or control IgG **(E)**. The area under the curve of exposure to Ara R **(F)**. Statistical analysis (mean ± SEM) with one-way ANOVA **(C, D)**. n = 6 from 2 independent experiments. The signs for statistical significance are p ≤ 0.05 (*), p ≤ 0.001 (***), and p ≤ 0.0001 (****).

Given that anti-IgE IgG antibodies can regulate serum IgE levels and basophil sensitization, we investigated their ability to protect mice from allergen challenges after IgE passive sensitization. Therefore, we first investigated whether passive immunization with anti-IgE IgG, anti-IgE(PNG) IgG, or a control IgG prior to IgE sensitization can protect against the Fel d 1 challenge (**Figure 3A**). Mice receiving anti-IgE IgG were protected from the Fel d 1 challenge, whereas this was not the case for mice immunized with the control IgG (**Figure 3B**). Immunization of mice with anti-IgE(PNG) IgG also conferred protection compared to the control group. However, comparing the area under (AUC) (drop of body temperature over time) of all three groups shows that anti-IgE IgG antibodies are significantly better in protecting mice from Fel d 1 challenge compared to anti-IgE(PNG) IgG antibodies (**Figure 3C**).

To ensure that not any residual anti-Fel d 1 IgG antibodies mediate the protection of the anti-IgE IgG antibodies, the same experiment was performed with Ara R as an allergen (**Figure 3D**). The results showed the same protection as when Fel d 1 was used as allergen indicating that anti-IgE IgG antibodies confer protection. Anti-IgE IgG and anti-IgE(PNG) IgG could significantly better protect mice from the Ara R challenge than the control IgG. However, again, anti-IgE IgG was more protective than Anti-IgE(PNG) IgG (**Figures 3E, F**).

These results suggest that anti-IgE IgG antibodies mediate the protection from allergen-challenge by active immunization of IgE-Fel d 1 complexes. Furthermore, they indicate that IgE glycosylation impacts the formation of IgG antibodies, which are efficient in protecting mice from allergen challenges.

### 3.4 Role of FcγRIIb and CD23 in the protection from anaphylaxis and clearance of IgE-IgG complexes

**Figure 4:**
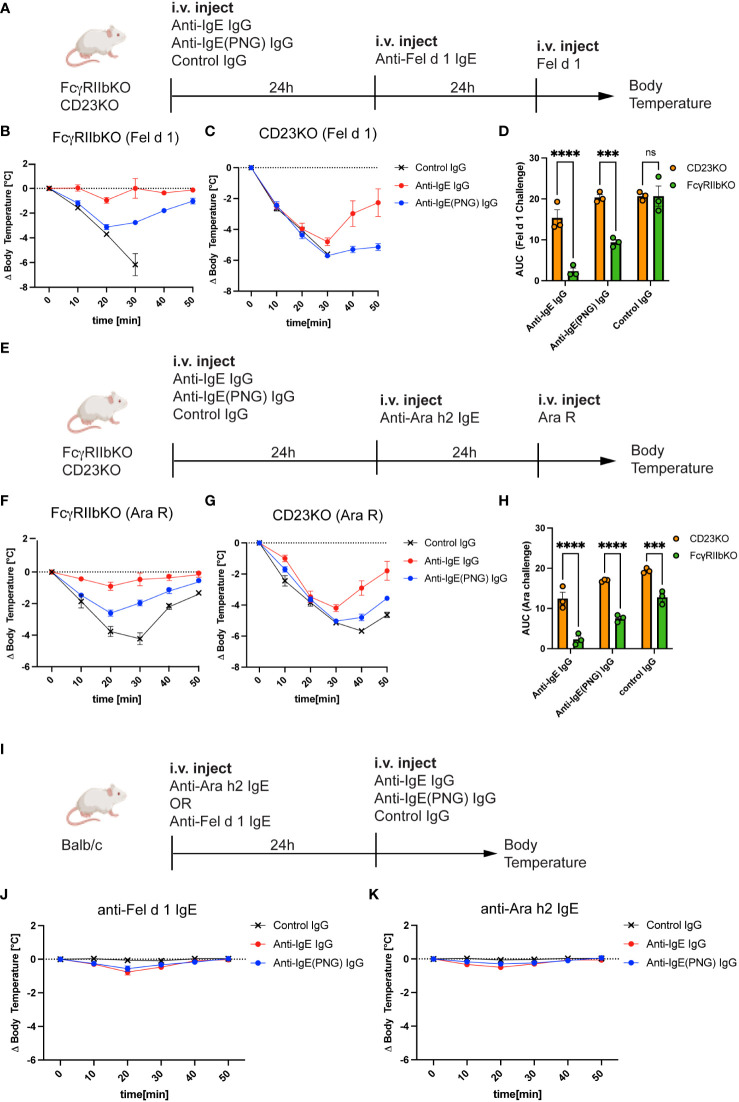
CD23 is important for the protection but not FcγRIIb. Schematic challenge overview with Fel d 1 and Ara R as an allergen, respectively, in FcγRIIbKO and CD23KO mice **(A, E)**. **(B, C)** Shows the decrease in body temperature (delta) over time following challenge with Fel d 1 after passive immunization with anti-IgE IgG, anti-IgE(PNG) IgG, or control IgG, in FcγRIIb KO **(B)** mice and in CD23KO mice **(C)**. AUC comparison between of Ara R challenged mice are displayed in **(D)**. Decrease in body temperature (delta) over time following challenge with Fel d 1 after passive immunization with anti-IgE IgG, anti-IgE(PNG) IgG, or control IgG, in FcγRIIb KO mice **(F)**, in CD23KO mice **(G)**. AUC comparison between of Fel d 1 challenged mice are displayed in **(H)**. Experimental setup to analyze the anaphylactic potential of the anti-IgE IgG antibodies **(I)**. **(J, K)** show the drop of bodytemperature of mice sensitized with anti-Fel d 1 **(H)** or anti-Ara h2 **(I)** IgE and challenged with the anti-IgE IgG antibodies. The signs for statistical significance are p ≤ 0.001 (***), and p ≤ 0.0001 (****).

Having shown that FcγRIIb and CD23 play a role in the binding of IgE-IC, we investigated the role of FcγRIIb and CD23 in the IgE clearance process and protection against anaphylaxis. For this purpose, we used FcγRIIbKO and CD23KO mice and passively sensitized them to Fel d 1 (**Figure 4A**) and Ara R (**Figure 4E**) after the passive immunization with the anti-IgE IgG antibodies. While FcγRIIbKO mice were still protected from subsequent challenges with Fel d 1 and Ara R (**Figures 4B, F**, respectively), CD23KO mice were not (**Figures 4C, G**). This suggests that protection is primarily mediated by neutralization and is independent of FcγRIIb. Comparing the AUC shows that FcγRIIB mice are significantly better protected from the Ara R challenge (**Figure 4D**) and the Fel d 1 challenge (**Figure 4H**) than CD23KO mice.

Next, we investigated whether anti-IgE IgG antibodies elicit an anaphylactic respons in IgE sensitized mice (**Figure 4I**) using IgE anti-Fel d 1 (**Figure 4J**) or IgE anti-Ara h 2 (**Figure 4K**). For this purpose, Balb/c mice were first injected with IgE and 24 hours later with the IgG antibodies and systemic anaphylaxis was measured by body core temperature. Both mouse groups were protected against anaphylaxis indicating that anti-IgE IgG antibodies induced by IgE-IC immunization do not cross-link FcϵRI bound IgE.

These results suggest that the inhibitory FcγRIIb is not involved in mediating protection from allergen challenge, but the low-affinity IgE receptor CD23.

### 3.5 Clearance of IgE-IgG complexes is mainly mediated by CD23

**Figure 5:**
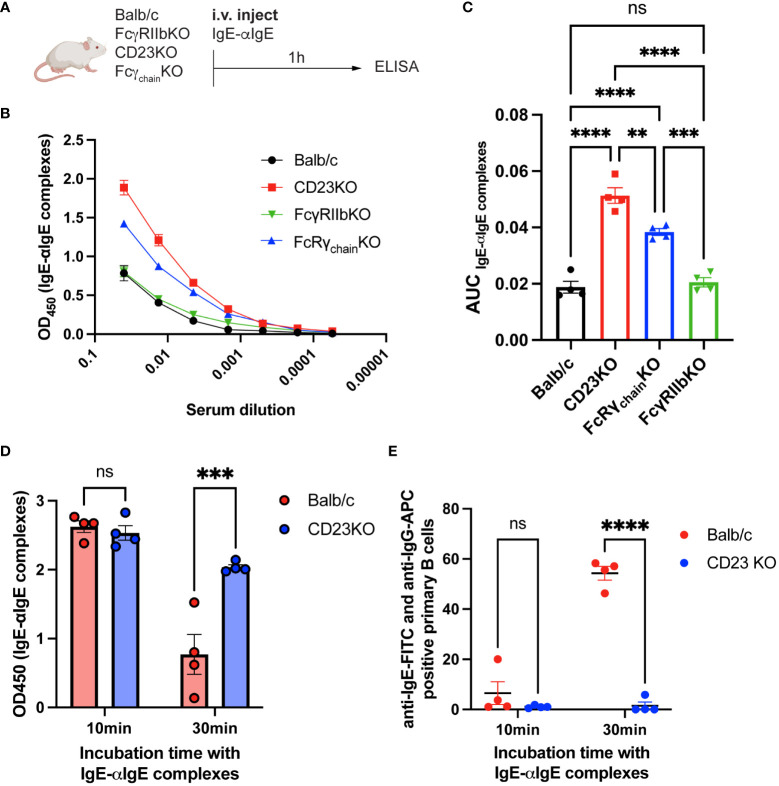
IgE-IgG complexes are mainly eliminated by CD23. Experimental overview for the clearance of IgE-anti-IgE IgG immune complexes **(A)**. IgE-anti-IgE IgG serum levels 1h after injection are shown as ELISA OD450 results **(B)** and as the area under the curve of the ELISA plot **(C)**. ELISA results of supernatant from IgE-anti-IgE IgG incubated with primary mouse B cells after 10min and 30min **(D)**. FACS results of anti-IgE and anti-IgG positive primary mouse B cells after 10min and 30min incubation with IgE-anti-IgE IgG immune complexes **(E)**. Statistical analysis (mean ± SEM) using one-way ANOVA **(C)** and two-way ANOVA **(D**, **E)**. n = 4 per group. The signs for statistical significance are ns = not significant, p ≤ 0.01 (**), p ≤ 0.001 (***), and p ≤ 0.0001 (****).

Since CD23KO mice were not protected, we hypothesized that IgE-IgG complexes might be eliminated *via* CD23. Thus, we investigated the influence of FcγRIIb, CD23, and Fcγ receptors on eliminating IgE-anti-IgE IgG complexes. Therefore, we immunized Balb/c, CD23KO, FcγRIIbKO, and common Fcγ-chainKO mice (lacking FcεRI, FcγRI, and FcγRIIIa) with IgE-anti-IgE IgG complexes (**Figure 5A**) and analyzed sera by ELISA 1h after injection. IgE-IgG complexes were still detected in CD23KO mice as well as to a lesser extent in Fcγ−chain KO mice. Significantly fewer IgE-IgG complexes were detected in Balb/c, FcγRKO and FcγRIIbKO mice (**Figures 5B, C**). To confirm these results, we isolated primary B cells from Balb/c and CD23 KO mice and incubated them with IgE-anti-IgE IgG complexes. The presence of complexes in supernatant were analyzed after 10 and 30 min by ELISA (**Figure 5D**). After 30 min incubation significantly fewer complexes were detected in the supernatant of B cells from WT mice than from CD23 KO mice. In parallel, we also assessed B cell surface complexes in these mice (**Figure 5E**). In line with the data from the cell supernatant data, B cells of wild type mice carried significantly more complexes than those of CD23 KO mice. These results suggest that CD23 and, to a lesser extent, FcγR mediate the elimination of IgE-IgG complexes, whereas FcγRIIb alone is not significantly involved in the clearance process.

## 4 Discussion

Targeting IgE with therapeutic anti-IgE antibodies such as omalizumab is a validated strategy to treat IgE-mediated diseases. Naturally occurring anti-IgE IgG antibodies have been identified in patients with various diseases, including asthma (37) and atopic dermatitis (38), but also in healthy individuals (39). Our recent work demonstrated that a single immunization with IgE-allergen complexes elicits an anti-IgE IgG response without adding adjuvants. The resulting anti-IgE IgG bind glycosylated IgE significantly better than deglycosylated IgE (33). Here we show that booster immunizations can even enhance the anti-IgE IgG response suggesting that repeated exposure to IgE-allergen IC might lead to a beneficial anti-IgE IgG response. Glycosylation of IgE plays a vital role in this process. The use of glycosylated IgE leads to an increased anti-IgE IgG and even an increased IgG anti-Fel d 1 response compared to deglycosylated IgE. The adjuvant effect of IgE for antigens has been previously demonstrated, but here we show that glycans are critical for this effect. The reduced immunogenicity of deglycosylated IgE might be counterintuitive. Removal of the glycans makes the IgE less soluble and could result in the formation of aggregates. It is known that aggregates can be important for antigenicity (40, 41). Hence, it is reasonable to assume that glycan-recognizing receptors are involved in the process. Shade et al. showed the impact of IgE glycosylation on pathogenicity. They showed that the conserved oligomannose at N394 for human IgE and N384 for mouse IgE is essential for binding to the FcεRI (42). Furthermore, they demonstrated that there are differences in IgE glycosylation between healthy and atopic individuals (43). While healthy individuals contain more N-acetylglucosamine and terminal galactose, peanut-allergic individuals showed a higher number of terminal sialic acids. Interestingly, removing sialic acid from IgE attenuates the degranulation of effector cells and, thereby, anaphylaxis. In contrast, the interaction between IgE and CD23 is glycan independent (44) and CD23KO mice show an even higher anti-IgE response (33). The fact that IgE is the most glycosylated immunoglobulin further strengthens this assumption. About 12% of its molecular weight is carbohydrate (11). Moreover, glycosylation of proteins influences their serum kinetic (45, 46). Fcγ-receptors in mice that bind IgE are FcγRIIb, FcγRIIIa, and FcγRIV (47). Specifically, for FcγRIV, one study has shown that this receptor binds IgE when IgE is present as a complex but not monomeric IgE (47). Moreover, the engagement of FcγRIV led to the antigen presentation to T cells (48) However, whether glycosylation of IgE plays a role in this process is unclear. Interestingly, two amino acids (K117 and E132) essential for FcεRI binding to IgE are conserved in FcγRIV (47, 49). Since IgE glycosylation is required for binding to FcεRI (13), it may also be necessary for binding to FcγRIV. One study investigated the effect of glycosylation of IgG-IC on binding to various human FcγRs. They showed that depending on the IgG subclass and Fcγ-receptor, PNGase F treated IgG-IC show reduced or no binding to the specific receptors anymore (50). Although the study was performed with human FcγR and IgG-IC, similar conditions might also apply to IgE-IC in mice. This would explain why more anti-IgE IgG was generated in the group with glycosylated IgE than in the group with deglycosylated IgE. It is also well known that Fcγ-receptors can induce T cell proliferation (51, 52). This would explain why we could not see a significant difference between glycosylated and deglycosylated IgE-IC in FcγR KO mice. Furthermore, it would explain why only IgE-IC, but not monomeric IgE, elicits an anti-IgE IgG response since FcγRs bind only complexed IgE (47, 53).

However, since there was also an anti-IgE IgG response in Fcγ-common chain KO mice, other receptors may also be involved in generating anti-IgE IgG antibodies upon IgE-IC immunization. A possible candidate could be the lectin galectin-9, which is known to bind IgE. Even though it has an anti-allergic effect by preventing IgE-antigen complex formation (54), it is not clear whether it can bind IgE-antigen complexes or not. However, it is known to induce DC maturation and promote T cell proliferation and Th1 cytokine production (55). However, since it binds IgE in a carbohydrate-dependent manner, we would expect to see differences between glycosylated and deglycosylated IgE-IC in the FcγKO group.

Further differences can be observed in the ability of anti-IgE IgG and anti-IgE(PNG) antibodies to clear IgE from serum and prevent binding to effector cells. The results show that anti-IgE IgG antibodies are significantly better at controlling the binding of IgE to FcεRI of basophils than anti-IgE(PNG) antibodies. This indicates that IgE glycosylation affects the quantity of anti-IgE antibodies generated and their quality. An explanation for this could be the difference in affinity since it is higher for the anti-IgE IgG antibodies than anti-IgE(PNG) antibodies. It could be that glycans are essential for interacting with anti-IgE IgG to IgE. Thus, in the case of missing glycans, the anti-IgE IgG binding site could be altered, resulting in decreased affinity. Another explanation could be that deglycosylation leads to a conformational change (13). This change would result in different conformational epitopes between glycosylated and deglycosylated IgE.

Passive immunization with anti-IgE IgG and anti-IgE(PNG) IgG allowed us to see if the difference in serum clearance is also reflected in the ability to protect mice from anaphylaxis upon allergen challenges. Our results show that both anti-IgE IgG antibodies protect BALB/c WT mice compared with controls. However, protection was always better in the anti-IgE IgG group than in the anti-IgE(PNG) group. These results are consistent with the clearance experiment results, where significantly more IgE was found on the basophils of the anti-IgE(PNG) IgG group than in those of the anti-IgE IgG group. To better understand the protection mechanism, allergen challenges were also performed in FcγRIIb KO and CD23 KO mice. Previous studies have shown that co-aggregation of FcγRIIb with FcεRI inhibits IgE-mediated degranulation of effector cells (56). Moreover, it has been observed that allergen-specific desensitization (SIT) induced allergen-specific IgGs, which act *via* FcγRIIb (34). However, since the FcγRIIbKO mice were as protected as the WT mice, we speculate that the protection is mainly mediated by the neutralization of IgE, primeraly *via* CD23 and FcγRs. In both B cells and monocyte-derived DCs, CD23 was shown to bind and internalize IgE-allergen complexes better than IgE alone (57) and could therefore also play a role in the clearance of IgE-anti-IgE IgG the complexes. Indeed, CD23KO mice were not as well protected as wild-type Balb/c mice, suggesting that CD23 is important for the clearance of IgE-IgG complexes. Furthermore, our serum kinetic results in CD23KO confirmed that most complexes are eliminated *via* CD23. Not enough elimination of complexes leads to the accumulation of IgE-IgG complexes and then to their dissociation, during which the released IgE can bind preferentially to FcεRI because of its higher affinity for FcεRI than for the IgG antibodies. This results in the sensitization of effector cells, which can degranulate upon encountering the allergen. Additionally, while IgE-ICs do not trigger empty FcεRI-mediated degranulation, IgE pre-sensitized basophils/mast cells can be degranulated by IgE-ICs (31). A lack of clearance could thus favor the degranulation of IgE pre-sensitized effector cells. FcγR might also play a role in the clearance of immune complexes. Indeed, several studies has reported FcγR for elimination of IgG complexes. Thus, IgE-omalizumab complexes has been shown to be cleared *via* interactions with FcγR on endothelial cells of the reticuloendothelial system (23). However, whether Fcγ-common chain KO mice respond similarly to allergen challenge as CD23KO mice cannot be verified since they also lack FcεRI, as FcεRI also relies on the common γ-chain.

In conclusion, we have demonstrated the importance of IgE glycosylation for the anti-IgE IgG response and a possible pathway to eliminate IgG-IgE complexes *via* CD23 in a mouse model. Further research is needed to investigate the effects of IgE glycosylation on anti-IgE IgG antibodies and serum IgE levels in humans. In particular, the extent to which different IgE glycosylation patterns affect anti-IgE IgG antibodies should also be investigated. More insights into the role of IgE glycosylation for anti-IgE IgG antibodies could help to find even more efficient strategies against type I hypersensitivity diseases.

## Data availability statement

The raw data supporting the conclusions of this article will be made available by the authors, without undue reservation.

## Ethics statement

The animal study was reviewed and approved by Swiss Federal Veterinary Office.

## Author contributions

KP designed, performed, and interpreted experiments. ZG and SZ helped perform experiments. MB, PE, and MV supervised, designed, and interpreted experiments. KP and MV wrote the manuscript. All authors contributed to the article and approved the submitted version.

## Funding

This project was supported by funding from the following grants: SNF grant 310030_179165/1 to MV; SNF grant 310039_185114 to MB.

## Acknowledgments

We acknowledge Marianne Zwicker and Aleksandra Nonic for technical assistance. Further, we thank Professor Jeffrey Ravetch for providing CD23 knockout mice.

## Conflict of interest

MB has a financial relationship with Saiba AG involving stock ownership or payments for research activities.

The remaining authors declare that the research was conducted in the absence of any commercial or financial relationships that could be construed as a potential conflict of interest.

## Publisher’s note

All claims expressed in this article are solely those of the authors and do not necessarily represent those of their affiliated organizations, or those of the publisher, the editors and the reviewers. Any product that may be evaluated in this article, or claim that may be made by its manufacturer, is not guaranteed or endorsed by the publisher.

## References

[1] Abraham SN St John AL. Mast cell-orchestrated immunity to pathogens. Nat Rev Immunol (2010) 10(6):440–52. doi: 10.1038/nri2782 PMC4469150 20498670

[2] Kraft S Kinet JP. New developments in FcepsilonRI regulation, function and inhibition. Nat Rev Immunol (2007) 7(5):365–78. doi: 10.1038/nri2072 17438574

[3] Kinet JP. The high-affinity IgE receptor (Fc epsilon RI): from physiology to pathology. Annu Rev Immunol (1999) 17:931–72. doi: 10.1146/annurev.immunol.17.1.931 10358778

[4] Weinberg EG. The WAO white book on allergy 2011-2012. Curr Allergy Clin Immunol (2011) 24(3):156–7.

[5] Lawrence MG Woodfolk JA Schuyler AJ Stillman LC Chapman MD Platts-Mills TAE. Half-life of IgE in serum and skin: Consequences for anti-IgE therapy in patients with allergic disease. J Allergy Clin Immunol (2017) 139(2):422–28.e4. doi: 10.1016/j.jaci.2016.04.056 27496596 PMC5405770

[6] Haba S Ovary Z Nisonoff A. Clearance of IgE from serum of normal and hybridoma-bearing mice. J Immunol (1985) 134(5):3291–7. 3980994

[7] Greer AM Wu N Putnam AL Woodruff PG Wolters P Kinet JP Shin JS. Serum IgE clearance is facilitated by human FcϵRI internalization. J Clin Invest. (2014) 124(3):1187–98. doi: 10.1172/JCI68964 PMC3938266 24569373

[8] Cheng LE Wang ZE Locksley RM. Murine b cells regulate serum IgE levels in a CD23-dependent manner. J Immunol (2010) 185(9):5040–7. doi: 10.4049/jimmunol.1001900 PMC3085987 20870945

[9] Yu P Kosco-Vilbois M Richards M Köhler G Lamers MC. Negative feedback regulation of IgE synthesis by murine CD23. Nature. (1994) 369(6483):753–6. doi: 10.1038/369753a0 8008068

[10] Fellmann M Buschor P Röthlisberger S Zellweger F Vogel M. High affinity targeting of CD23 inhibits IgE synthesis in human b cells. Immun Inflammation Dis (2015) 3(4):339–49. doi: 10.1002/iid3.72 PMC4693728 26732048

[11] Shade KT Conroy ME Anthony RM. IgE glycosylation in health and disease. Curr Top Microbiol Immunol (2019) 423:77–93. doi: 10.1007/82_2019_151 30820668 PMC6750212

[12] Shade KTC Conroy ME Washburn N Kitaoka M Huynh DJ Laprise E. Sialylation of immunoglobulin e is a determinant of allergic pathogenicity. Nature. (2020) 582(7811):265–70. doi: 10.1038/s41586-020-2311-z PMC7386252 32499653

[13] Shade KTC Platzer B Washburn N Mani V Bartsch YC Conroy M A single glycan on IgE is indispensable for initiation of anaphylaxis. J Exp Med (2015) 212(4):457–67. doi: 10.1084/jem.20142182 PMC4387292 25824821

[14] Braunstahl GJ Chen CW Maykut R Georgiou P Peachey G Bruce J. The eXpeRience registry: the “real-world” effectiveness of omalizumab in allergic asthma. Respir Med (2013) 107(8):1141–51. doi: 10.1016/j.rmed.2013.04.017 23721684

[15] Abraham I Alhossan A Lee CS Kutbi H MacDonald K. “Real-life” effectiveness studies of omalizumab in adult patients with severe allergic asthma: Systematic review. Allergy. (2016) 71(5):593–610. doi: 10.1111/all.12815 26644231

[16] Saini SS Bindslev-Jensen C Maurer M Grob JJ Bülbül Baskan E Bradley MS. Efficacy and safety of omalizumab in patients with chronic Idiopathic/Spontaneous urticaria who remain symptomatic on H1 antihistamines: A randomized, placebo-controlled study. J Invest Dermatol (2015) 135(3):925. doi: 10.1038/jid.2014.512 25501032

[17] Lanier B Bridges T Kulus M Taylor AF Berhane I Vidaurre CF. Omalizumab for the treatment of exacerbations in children with inadequately controlled allergic (IgE-mediated) asthma. J Allergy Clin Immunol (2009) 124(6):1210–6. doi: 10.1016/j.jaci.2009.09.021 19910033

[18] Maurer M Rosén K Hsieh HJ Saini S Grattan C Gimenéz-Arnau A. Omalizumab for the treatment of chronic idiopathic or spontaneous urticaria. N Engl J Med (2013) 368(10):924–35. doi: 10.1056/NEJMoa1215372 23432142

[19] Lelegren MJ Son SY Han JK Lam KK. A review of phase III clinical trials of US FDA-approved biologic therapies for chronic rhinosinusitis with nasal polyposis. Immunotherapy. (2022) 14(8):655–62. doi: 10.2217/imt-2021-0310 35510314

[20] Ayats-Vidal R Riera-Rubió S Valdesoiro-Navarrete L García-González M Larramona-Carrera H Cruz OA. Long-term outcome of omalizumab-assisted desensitisation to cow’s milk and eggs in patients refractory to conventional oral immunotherapy: real-life study. Allergol Immunopathol (Madr). (2022) 50(3):1–7. doi: 10.15586/aei.v50i3.537 35527650

[21] Nadeau KC Kohli A Iyengar S DeKruyff RH Umetsu DT. Oral immunotherapy and anti-IgE antibody-adjunctive treatment for food allergy. Immunol Allergy Clin North Am (2012) 32(1):111–33. doi: 10.1016/j.iac.2011.11.004 22244236

[22] Lieberman JA Chehade M. Use of omalizumab in the treatment of food allergy and anaphylaxis. Curr Allergy Asthma Rep (2013) 13(1):78–84. doi: 10.1007/s11882-012-0316-x 23065311

[23] Lowe PJ Tannenbaum S Gautier A Jimenez P. Relationship between omalizumab pharmacokinetics, IgE pharmacodynamics and symptoms in patients with severe persistent allergic (IgE-mediated) asthma. Br J Clin Pharmacol (2009) 68(1):61–76. doi: 10.1111/j.1365-2125.2009.03401.x 19660004 PMC2732941

[24] Chan YC Ramadani F Santos AF Pillai P Ohm-Laursen L Harper CE. “auto-anti-IgE”: Naturally occurring IgG anti-IgE antibodies may inhibit allergen-induced basophil activation. J Allergy Clin Immunol (2014) 134(6):1394–1401.e4. doi: 10.1016/j.jaci.2014.06.029 25112697 PMC4258608

[25] Magnusson CG. Naturally occurring human IgA autoantibodies against IgE-DES myeloma protein. Prevalence specificity. Allergy (1994) 49(10):820–6. doi: 10.1111/j.1398-9995.1994.tb00781.x 7535980

[26] Galeotti C Karnam A Dimitrov JD Chevailler A Kaveri SV Bayry J. Anti-IgE IgG autoantibodies isolated from therapeutic normal IgG intravenous immunoglobulin induce basophil activation. Cell Mol Immunol (2020) 17(4):426–9. doi: 10.1038/s41423-019-0334-x PMC7109030 31797906

[27] Marshall JS Bell EB. Induction of an auto-anti-IgE response in rats i. effects on serum IgE concentrations. Eur J Immunol (1985) 15(3):272–7. doi: 10.1002/eji.1830150312 3872217

[28] Marshall JS Bell EB. Induction of an auto-anti-IgE response in rats. III. inhibition of a specific IgE response. Immunology. (1989) 66(3):428–33. PMC1385232 2784782

[29] Haba S Nisonoff A. Inhibition of IgE synthesis by anti-IgE: role in long-term inhibition of IgE synthesis by neonatally administered soluble IgE. Proc Natl Acad Sci U S A. (1990) 87(9):3363–7. doi: 10.1073/pnas.87.9.3363 PMC53900 2185467

[30] Haba S Nisonoff A. Effects of syngeneic anti-IgE antibodies on the development of IgE memory and on the secondary IgE response. J Immunol (1994) 152(1):51–7. 8254205

[31] Engeroff P Caviezel F Mueller D Thoms F Bachmann MF Vogel M. CD23 provides a noninflammatory pathway for IgE-allergen complexes. J Allergy Clin Immunol (2020) 145(1):301–311.e4. doi: 10.1016/j.jaci.2019.07.045 31437490

[32] Stanworth DR Jones VM Lewin IV Nayyar S. Allergy treatment with a peptide vaccine. Lancet. (1990) 336(8726):1279–81. doi: 10.1016/0140-6736(90)92963-i 1700248

[33] Engeroff P Plattner K Storni F Thoms F Frias Boligan K Muerner L. Glycan-specific IgG anti-IgE autoantibodies are protective against allergic anaphylaxis in a murine model. J Allergy Clin Immunol (2021) 147(4):1430–41. doi: 10.1016/j.jaci.2020.11.031 33309740

[34] Uermösi C Beerli RR Bauer M Manolova V Dietmeier K Buser RB. Mechanisms of allergen-specific desensitization. J Allergy Clin Immunol (2010) 126(2):375–83. doi: 10.1016/j.jaci.2010.05.040 20624641

[35] Croote D Darmanis S Nadeau KC Quake SR. High-affinity allergen-specific human antibodies cloned from single IgE b cell transcriptomes. Science. (2018) 362(6420):1306–9. doi: 10.1126/SCIENCE.AAU2599 30545888

[36] Koppelman SJ Jayasena S Luykx D Schepens E Apostolovic D de Jong GA. Allergenicity attributes of different peanut market types. Food Chem Toxicol (2016) 91:82–90. doi: 10.1016/j.fct.2016.02.016 26921497

[37] Nawata Y Koike T Hosokawa H Tomioka H Yoshida S. Anti-IgE autoantibody in patients with atopic dermatitis. J Immunol (1985) 135(1):478–82. 3873495

[38] Swainson JA Wilson PB Dore P Pumphrey RSH. Evidence for circulating complexes containing IgE in patients with atopic dermatitis. Int Arch Allergy Appl Immunol (1985) 76(3):237–42. doi: 10.1159/000233698 3871737

[39] Ritter C Bättig M Kraemer R Stadler BM. IgE hidden in immune complexes with anti-IgE autoantibodies in children with asthma. J Allergy Clin Immunol (1991) 88(5):793–801. doi: 10.1016/0091-6749(91)90187-S 1955638

[40] Braun A Kwee L Labow MA Alsenz J. Protein aggregates seem to play a key role among the parameters influencing the antigenicity of interferon alpha (IFN-alpha) in normal and transgenic mice. Pharm Res (1997) 14(10):1472–8. doi: 10.1023/a:1012193326789 9358564

[41] Moore WV Leppert P. Role of aggregated human growth hormone (hGH) in development of antibodies to hGH. J Clin Endocrinol Metab (1980) 51(4):691–7. doi: 10.1210/jcem-51-4-691 7419661

[42] Shade KT Platzer B Washburn N Mani V Bartsch YC Conroy M. A single glycan on IgE is indispensable for initiation of anaphylaxis. J Exp Med (2015) 212(4):457–67. doi: 10.1084/jem.20142182 PMC4387292 25824821

[43] Shade KC Conroy ME Washburn N Kitaoka M Huynh DJ Laprise E. Sialylation of immunoglobulin E is a determinant of allergic pathogenicity. Nature (2020) 582(7811):265–70. doi: 10.1038/s41586-020-2311-z PMC7386252 32499653

[44] Vercelli D Helm B Marsh P Padlan E Geha RS Gould H. The b-cell binding site on human immunoglobulin e. Nature. (1989) 338(6217):649–51. doi: 10.1038/338649a0 2468089

[45] Jefferis R. Glycosylation as a strategy to improve antibody-based therapeutics. Nat Rev Drug Discovery (2009) 8:226. doi: 10.1038/nrd2804 19247305

[46] Nettleton MY Kochan JP. Role of glycosylation sites in the IgE fc molecule. Int Arch Allergy Immunol (1995) 107(1-3):328–9. doi: 10.1159/000237017 7613162

[47] Mancardi DA Iannascoli B Hoos S England P Daëron M Bruhns P. FcγRIV is a mouse IgE receptor that resembles macrophage FcϵRI in humans and promotes IgE-induced lung inflammation. J Clin Invest. (2008) 118(11):3738. doi: 10.1172/JCI36452 18949059 PMC2571035

[48] Hirano M Davis RS Fine DW Nakamura S Shimizu K Yagi H. IgEb immune complexes activate macrophages through FcgammaRIV binding. Nat Immunol (2007) 8(7):762–71. doi: 10.1038/NI1477 17558411

[49] Garman SC Wurzburg BA Tarchevskaya SS Kinet JP Jardetzky TS. Structure of the fc fragment of human IgE bound to its high-affinity receptor fc epsilonRI alpha. Nature (2000) 406(6793):259–66. doi: 10.1038/35018500 10917520

[50] Lux A Yu X Scanlan CN Nimmerjahn F. Impact of immune complex size and glycosylation on IgG binding to human FcγRs. J Immunol (2013) 190(8):4315–23. doi: 10.4049/JIMMUNOL.1200501 23509345

[51] Junker F Gordon J Qureshi O. Fc gamma receptors and their role in antigen uptake, presentation, and T cell activation. Front Immunol (2020) 11:1393. doi: 10.3389/FIMMU.2020.01393 32719679 PMC7350606

[52] Yada A Ebihara S Matsumura K Endo S Maeda T Nakamura A. Accelerated antigen presentation and elicitation of humoral response *in vivo* by FcγRIIB- and FcγRI/III-mediated immune complex uptake. Cell Immunol (2003) 225(1):21–32. doi: 10.1016/J.CELLIMM.2003.09.008 14643301

[53] Takizawa F Adamczewski M Kinet JP. Identification of the low affinity receptor for immunoglobulin e on mouse mast cells and macrophages as fc gamma RII and fc gamma RIII. J Exp Med (1992) 176(2):469–76. doi: 10.1084/JEM.176.2.469 PMC2119311 1386873

[54] Niki T Tsutsui S Hirose S Aradono S Sugimoto Y Takeshita K. Galectin-9 is a high affinity IgE-binding lectin with anti-allergic effect by blocking IgE-antigen complex formation. J Biol Chem (2009) 284(47):32344–52. doi: 10.1074/jbc.M109.035196 PMC2781649 19776007

[55] Dai SY Nakagawa R Itoh A Murakami H Kashio Y Abe H. Galectin-9 induces maturation of human monocyte-derived dendritic cells. J Immunol (2005) 175(5):2974–81. doi: 10.4049/jimmunol.175.5.2974 16116184

[56] Ekoff M Möller C Xiang Z Nilsson G. Coaggregation of FcepsilonRI with FcgammaRIIB inhibits degranulation but not induction of bcl-2 family members A1 and bim in mast cells. Allergy Asthma Clin Immunol (2006) 2(3):87–97. doi: 10.1186/1710-1492-2-3-87 20525153 PMC2876181

[57] Engeroff P Fellmann M Yerly D Bachmann MF Vogel M. A novel recycling mechanism of native IgE-antigen complexes in human b cells facilitates transfer of antigen to dendritic cells for antigen presentation. J Allergy Clin Immunol (2018) 142(2):557–68.e6. doi: 10.1016/j.jaci.2017.09.024 29074459

