# Supplementary figures and images for: IgE glycans promote anti-IgE IgG autoantibodies that facilitate IgE serum clearance via Fc Receptors
Source: Front Immunol. 2022 Dec 2;13:1069100. doi: 10.3389/fimmu.2022.1069100 (PMC9761184; doi:10.3389/fimmu.2022.1069100)

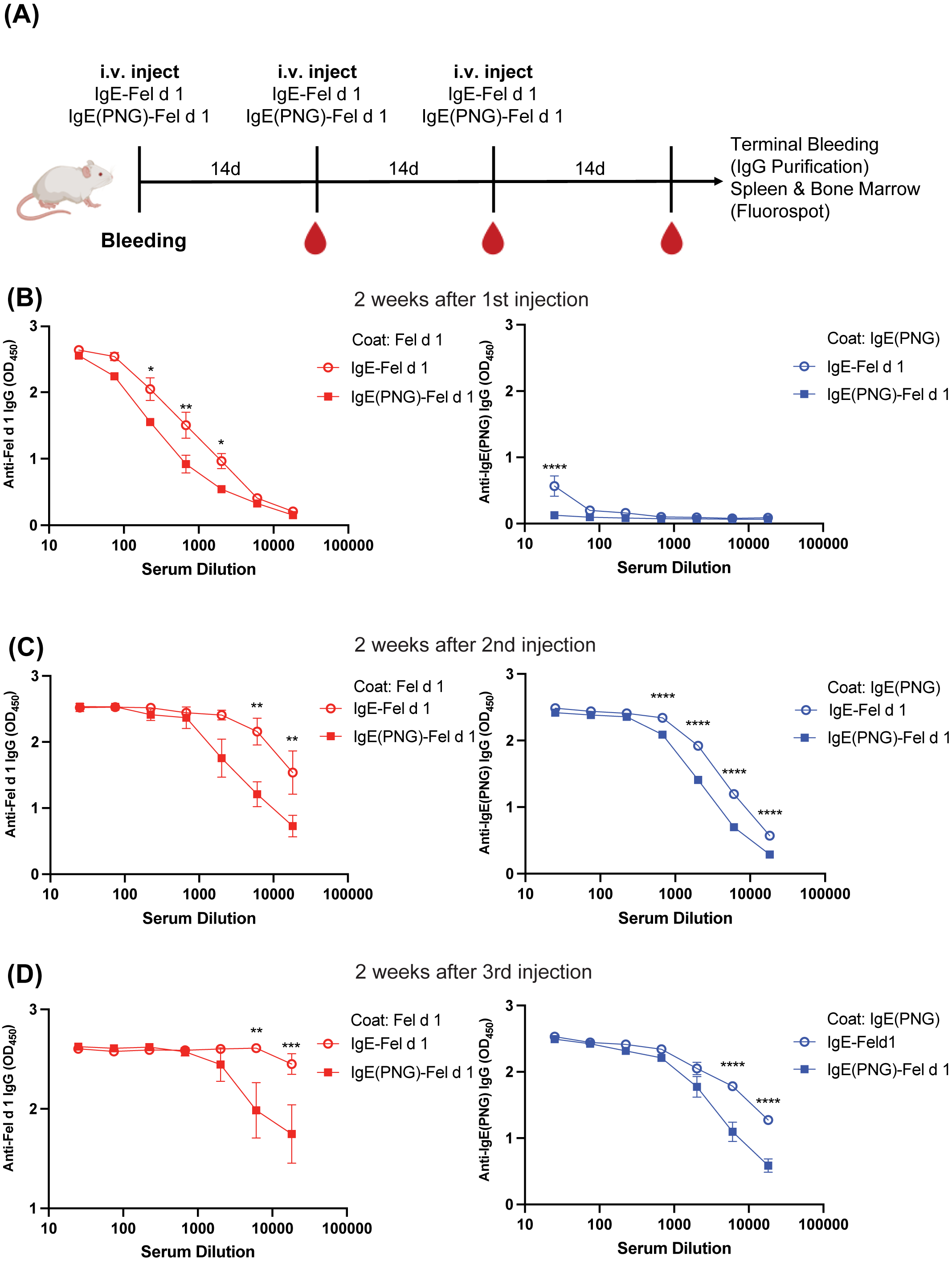

Supplement: Supplementary file 1 [file Image_1.tif]

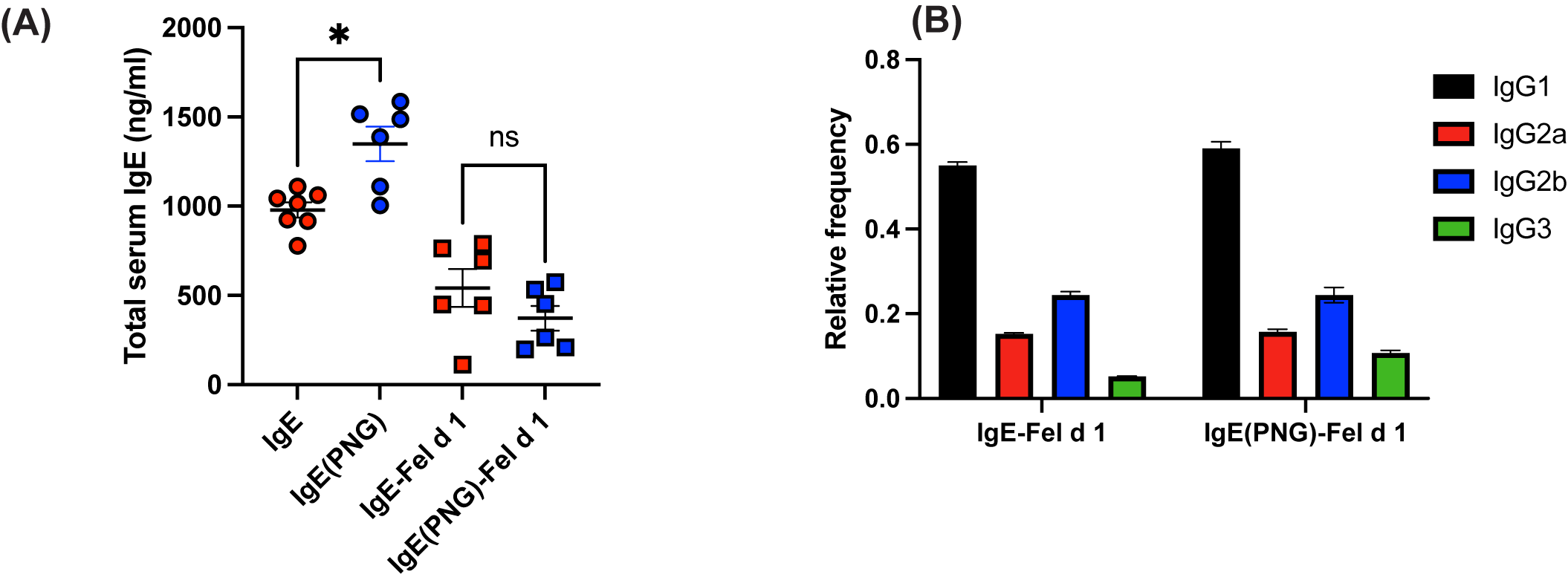

Supplement: Supplementary file 2 [file Image_2.tif]

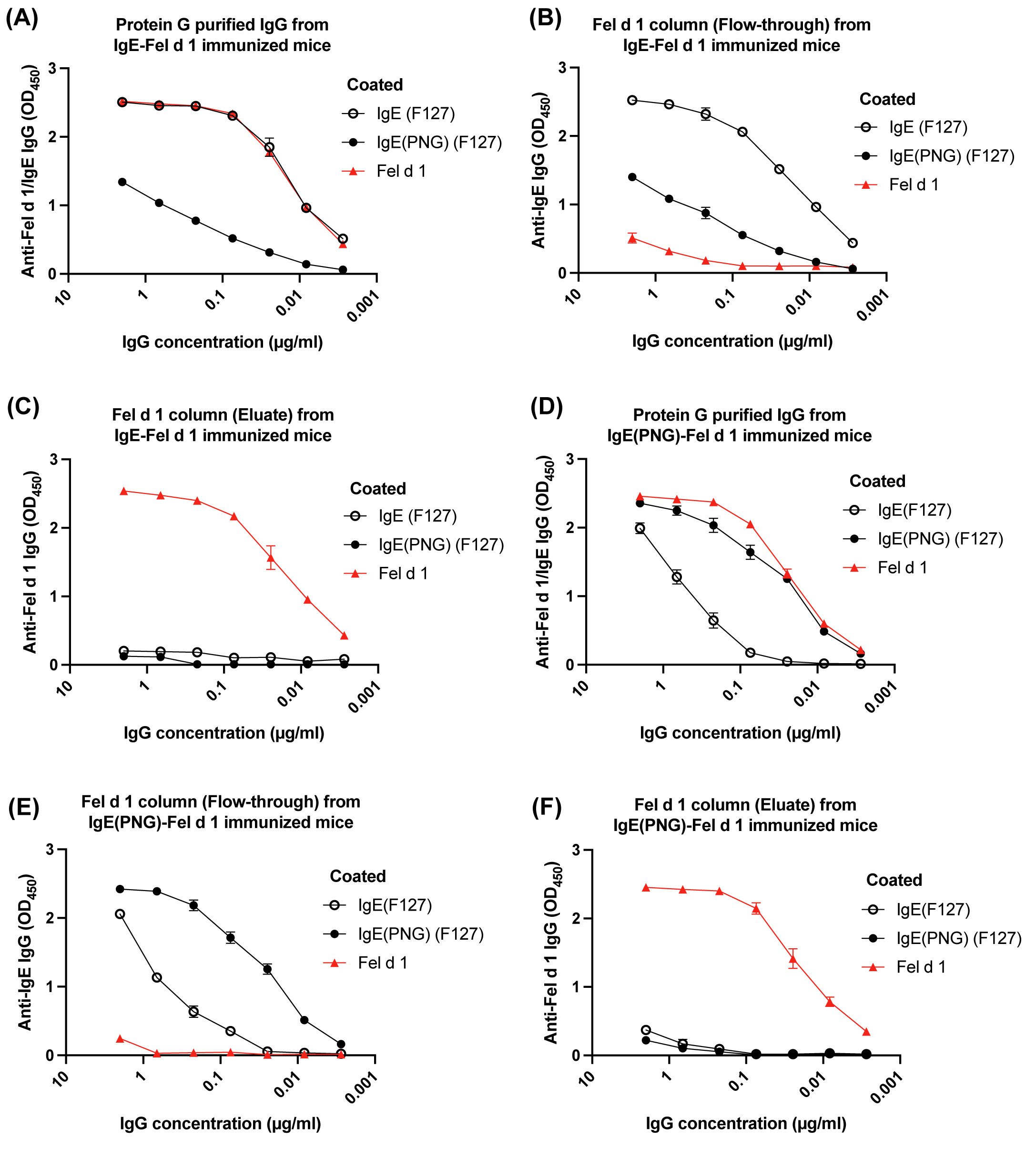

Supplement: Supplementary file 3 [file Image_3.tif]
